# TAT peptide at treatment-level concentrations crossed brain endothelial cell monolayer independent of receptor-mediated endocytosis or peptide-inflicted barrier disruption

**DOI:** 10.1371/journal.pone.0292681

**Published:** 2023-10-11

**Authors:** Meng-Chih Wu, Eric Yuhsiang Wang, Ted Weita Lai

**Affiliations:** 1 Graduate Institute of Biomedical Sciences, China Medical University, Taichung, Taiwan; 2 School of Chinese Medicine, China Medical University, Taichung, Taiwan; 3 School of Medicine, China Medical University, Taichung, Taiwan; 4 Neuroscience and Brain Disease Center, China Medical University, Taichung, Taiwan; 5 Drug Development Center, China Medical University, Taichung, Taiwan; 6 Translational Medicine Research Center, China Medical University Hospital, Taichung, Taiwan; University of Michigan Medical School, UNITED STATES

## Abstract

The peptide domain extending from residues 49 to 57 of the HIV-1 Tat protein (TAT) has been widely shown to facilitate cell entry of and blood-brain barrier (BBB) permeability to covalently bound macromolecules; therefore, TAT-linked therapeutic peptides trafficked through peripheral routes have been used to treat brain diseases in preclinical and clinical studies. Although the mechanisms underlying cell entry by similar peptides have been established to be temperature-dependent and cell-type specific and to involve receptor-mediated endocytosis, how these peptides cross the BBB remains unclear. Here, using an *in vitro* model, we studied the permeability of TAT, which was covalently bound to the fluorescent probe fluorescein isothiocyanate (FITC), and evaluated whether it crossed the “*in vitro* BBB”, a monolayer of brain endothelial cells, and whether the mechanisms were similar to those involved in TAT entry into cells. Our results show that although TAT crossed the monolayer of brain endothelial cells in a temperature-dependent manner, in contrast to the reported mechanism of cell entry, it did not require receptor-mediated endocytosis. Furthermore, we revisited the hypothesis that TAT facilitates brain delivery of covalently bound macromolecules by causing BBB disruption. Our results demonstrated that the dose of TAT commonly used in preclinical and clinical studies did not exert an effect on BBB permeability *in vitro* or *in vivo*; however, an extremely high TAT concentration caused BBB disruption *in vitro*. In conclusion, the BBB permeability to TAT is temperature-dependent, but at treatment-level concentrations, it does not involve receptor-mediated endocytosis or BBB disruption.

## Introduction

Tat is an 86 amino acid trans-activator protein encoded by HIV-1 that readily enters cells when incubated exogenously in culture medium [1, 2]. When chemically cross-linked or fused to proteins that are otherwise impermeable to cellular membrane barriers, Tat or synthetic fragments of Tat facilitates the delivery of these proteins into cultured cells *in vitro* and rodent tissues *in vivo* [3–10]. In addition, early analysis of truncated Tat peptide fragments has shown that its basic amino acid domain, extending from residues 49 to 57 (single-letter code, RKKRRQRRR), is sufficient for cell membrane translocation [11, 12]. Furthermore, consistent with the key role of arginine residues in this process, the amount of polyarginine oligopeptide (R^9^) taken up by cells is similar to or greater than that of peptides carrying the Tat^49-57^ domain [12–15]. In light of cell membrane and blood-brain barrier (BBB) permeability to these peptides, a growing number of Tat^49-57^-linked therapeutic peptides and proteins have been developed for experimental and clinical use.

Although peptides containing the Tat^49-57^ sequence or R^9^ are initially thought to exclusively penetrate cells in a temperature-, energy-, and receptor-independent manner [11, 13–15], later experiments demonstrated that these early observations were due to experimental artifacts related to cell fixation and flow cytometry analysis [16, 17]. Notably, several studies now support the notion that cellular uptake of these and related peptides administered at lower concentrations is energy- and temperature-dependent [16, 18–21], is cell-type-specific [22–24], and involves several different mechanisms of endocytosis [19–21, 23, 25–34]. Moreover, at higher concentrations and under certain experimental conditions, these peptides can directly penetrate into cells via non-endocytic mechanisms, in part by causing transient focal deformation of certain regions of the cell membrane [24, 29, 32, 35–40].

Contrary to the mechanisms of cellular uptake, the mechanism by which Tat-derived peptides cross the BBB has not been well characterized. Notably, several lines of evidence have shown that the full-length Tat protein can disrupt the BBB by increasing vascular endothelial cell permeability [41, 42], causing endothelial cell cytotoxicity [43–47], and modulating the expression and cellular localization of the tight junction proteins needed to maintain adhesion of brain endothelial cells to each other [48–52]. Accordingly, it has been postulated that Tat-derived peptides enter the brain by causing BBB disruption as well [53, 54]; however, experiments in this regard have led to inconsistent results [53, 54]. In this study, using an *in vitro* BBB model, we found that the short peptide TAT (amino acid sequence: YGRKKRRQRRR) crossed the BBB in a temperature-dependent manner, but did not share the same mechanisms by which Tat-derived peptides entered cells. Moreover, using an *in vivo* model, we showed that TAT, administered at a commonly used dose, could not increase BBB permeability.

## Materials and methods

### Cell culture

The bEnd.3 (mouse brain endothelial cell) cell line, purchased from Bioresource Collection and Research Center (BCRC, Taiwan), was used in this study. Prior to experiments, the cells were cultured in Dulbecco’s modified Eagle’s medium (DMEM; HyClone, cat. #SH30003.02) with 10% fetal bovine serum (Gibco, cat. #10437028) and 1% penicillin-streptomycin (Gibco, cat. #15140112), and were passaged every 2–3 days with 0.25% trypsin-EDTA (Gibco, cat. #25200056).

### Cellular metabolic activity

Cellular metabolic activity was measured by MTT (thiazolyl blue tetrazolium bromide) assay 24 h after treatment by normothermia (37°C), hypothermia (4°C), sodium azide (NaN_3_)/2-deoxy-D-glucose (2-DG), cytochalasin D, 5-(N-ethyl-N-isopropyl)-amiloride (EIPA), or methyl-beta-cyclodextrin (MβCD). In brief, MTT assay was performed with 2000 cells in culture by first replacing the cell culture medium with medium containing 0.5 mg/ml MTT (Alfa Aesar, cat. #L11939). After a 4-h incubation period to allow for MTT metabolism, the MTT-containing medium was replaced with 100 μl of dimethyl sulfoxide (DMSO; JT Baker), and the culture plate was placed on a shaker for 10 min to allow for dissolution of the violet crystal metabolites. Thereafter, the optical density of the metabolite was measured at 570 nm by an ELISA reader. The optical density of the metabolite in the hypothermia and drug treatment groups was expressed as percentage relative to the optical density of the metabolite in the normothermia and control groups, respectively.

### Permeability across a monolayer of brain endothelial cells

An *in vitro* BBB model composed of a monolayer of bEnd.3 cells was used to measure the permeability of the fluorescent probe FITC covalently bound to TAT and related peptides as described previously [55]. Briefly, the cells were seeded onto 24-well culture inserts (Falcon Permeable Support for 24-well plate; cat. #353104), and one-half of the medium was replaced every other day, and the cells were cultured for 5 days prior to the performance of a permeability assay. On the day of the assay, the culture medium was replaced with serum-free DMEM with FITC-linked peptides or bovine serum albumin (Sigma). The permeability of the FITC-linked peptides and albumin was measured for a total period of 12 or 24 h by collecting 50 μl of the medium from the lower compartment below the insert in each well and quantifying them by spectrophotometry and western blotting, respectively. Notably, the total period of diffusion was 12 h in the experiments where we compared hypothermia versus normothermia, because a substantial effect of hypothermia was observed by this early timepoint. To ensure a lack of effect, the total period of diffusion was extended to 24 h in other experiments, because a substantial effect was not observed after 12 h of diffusion.

### Mice and measurement of BBB *in vivo*

The BBB permeability in male C57BL/6 mice (8 weeks old; 25–35 g), which were assigned to either a control group and received vehicle injections (PBS, i.v. via tail vein), a group that received craniectomy, or a group that received TAT injections (3 nmol/g, dissolved in PBS, i.v. via tail vein), was determined as described previously [56]. Briefly, 24 h after craniectomy or a TAT injection, the mice were euthanized by an urethane (i.p.) overdose and were perfused with cold saline. The cortices of the right hemispheres were collected from coronal sections located 2–4 mm anterior to lambda, and the relative concentrations of albumin and tubulin within the brain tissue were measured by western blotting. All experimental procedures described herein were performed in accordance with ARRIVE guidelines and Institutional Guidelines for China Medical University (CMU) for the Care and Use of Experimental Animals and were approved by the Institutional Animal Care and Use Committee of CMU (protocol No. 103-224-NH).

### Western blotting

Medium collected from cell culture *in vitro* (12 μl medium per culture) and tissue samples harvested from mice *in vivo* were denatured by boiling in lysis buffer, separated by electrophoresis, transferred onto PVDF membranes, and imaged after incubation in primary and secondary antibodies as described previously for *in vitro* [55] and *in vivo* [57] experiments. The following primary antibodies were used: anti-albumin (1:2000; GeneTex, cat. #102419, for the *in vitro* experiments; 1:5000; Abcam, cat. #ab106582, for the *in vivo* experiments) and anti-ɑ-tubulin (1:5000; GeneTex, cat. #GTX628802) antibodies, and the following secondary antibodies were used: anti-rabbit (1:5000; GeneTex, cat. #GTX213110-01), anti-mouse (1:1000; GeneTex, cat. #GTX213111-01), and anti-chicken (1:1000; Abcam, cat. #ab97135) antibodies.

### Data presentation and statistical analysis

The data are presented as either the mean and individual data points or the mean ± SEM. Peptide diffusion over time was analyzed by performing 2-way repeated-measures ANOVA, matching peptide concentrations in the same culture medium over time, followed by Sidak’s multiple comparisons test. Cellular metabolic activity levels were compared by unpaired t test or 2-way ANOVA followed by Sidak’s multiple comparisons test. Albumin permeability *in vitro* and *in vivo* was determined by 1-way ANOVA followed by Sidak’s multiple comparisons test.

## Results

### Tat-derived peptide crosses a monolayer of brain endothelial cells in a temperature-dependent manner

**Fig 1:**
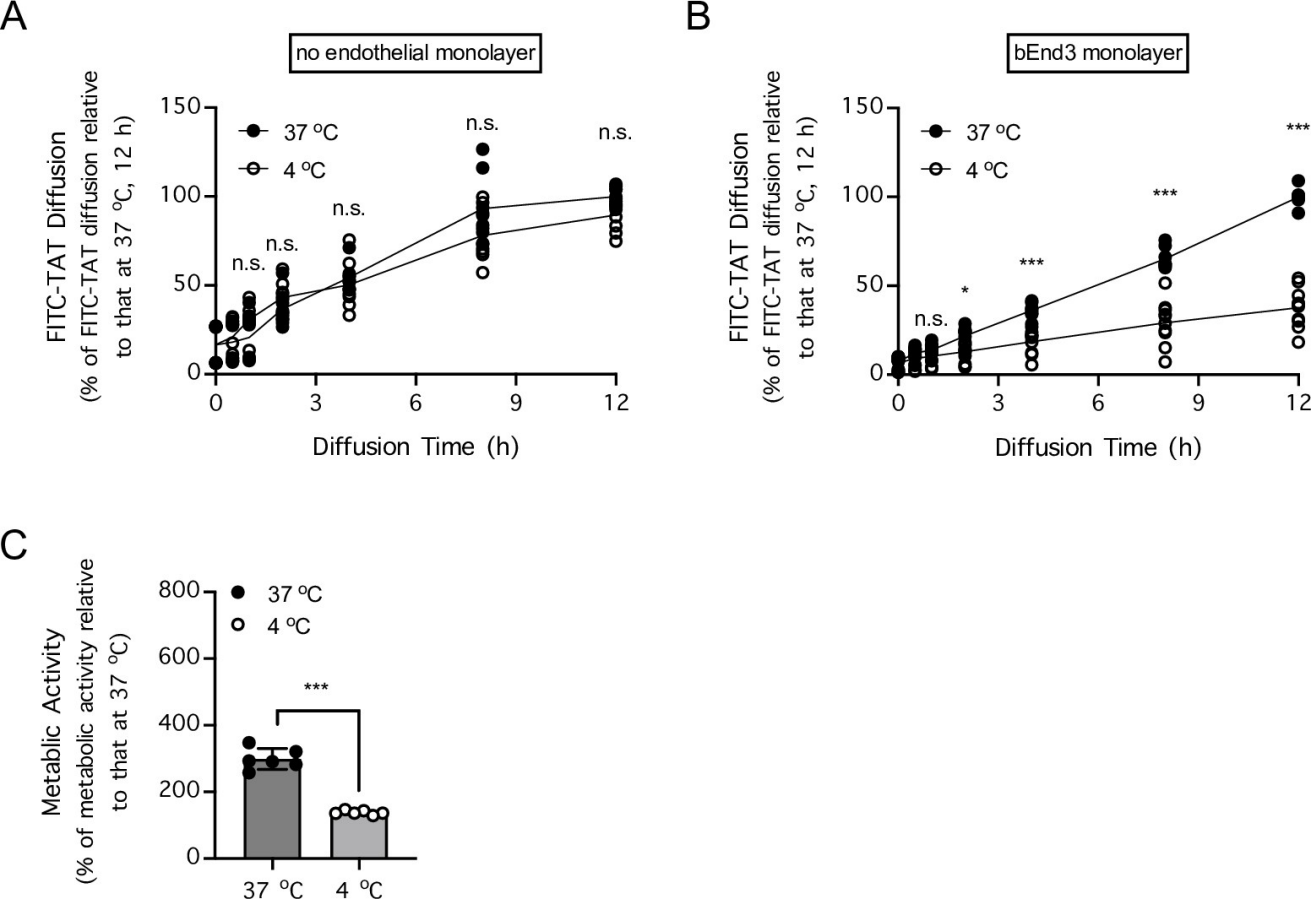
Diffusion of FITC-TAT across a monolayer of brain endothelial cells is temperature-dependent. ***A-B***, Diffusion of FITC-TAT (10 μM) under normothermic conditions (37°C; ●) or hypothermic conditions (4°C; ○) across Transwell membranes (pore diameter of 1 μm) without (***A***) or with (***B***) a monolayer composed of the bEnd.3 mouse brain endothelial cell line were measured 30 min and 1, 2, 4, 8, and 12 h post-diffusion onset via fluorescence spectrophotometry. n = 8–10 cell cultures per group. * *P* < 0.05, *** *P* < 0.001, when 4°C was compared to 37°C by 2-way repeated-measures ANOVA, matching [Tat-FITC] in the same culture medium over time followed by Sidak’s multiple comparisons test. n.s. indicates no significant difference. ***C***, Cellular metabolic activity levels of the bEnd.3 cells were measured by MTT assay under normothermic conditions (37°C; ●) or hypothermic conditions (4°C; ○). n = 6 cell cultures per group. *** *P* < 0.001 at 4°C compared to 37°C by unpaired t test.

When dissolved into the upper chamber of Transwell inserts, synthetic fluorescein isothiocyanate (FITC)-TAT (TAT with FITC covalently bound to the peptide N-terminus) readily diffused across to the lower chamber at 37°C, regardless of whether monolayers of brain endothelial cells were cultured on the Transwell membranes (Fig 1A and 1B). Importantly, lowering the temperature to 4°C had no effect on the diffusion of FITC-TAT across the Transwell inserts when the endothelial monolayers were absent (Fig 1A), but substantially decreased the diffusion of FITC-TAT across the Transwell inserts when the endothelial monolayers were present (Fig 1B). Consistent with the need for cellular metabolic activity for the endothelial cell monolayer permeability to FITC-TAT, lowering the temperature to 4°C decreased metabolism of the endothelial cells in culture (Fig 1C). These data are consistent with the notion that ability of FITC-TAT to cross a monolayer of brain endothelial cells relied on cellular metabolic activities that were temperature-dependent.

**Fig 2:**
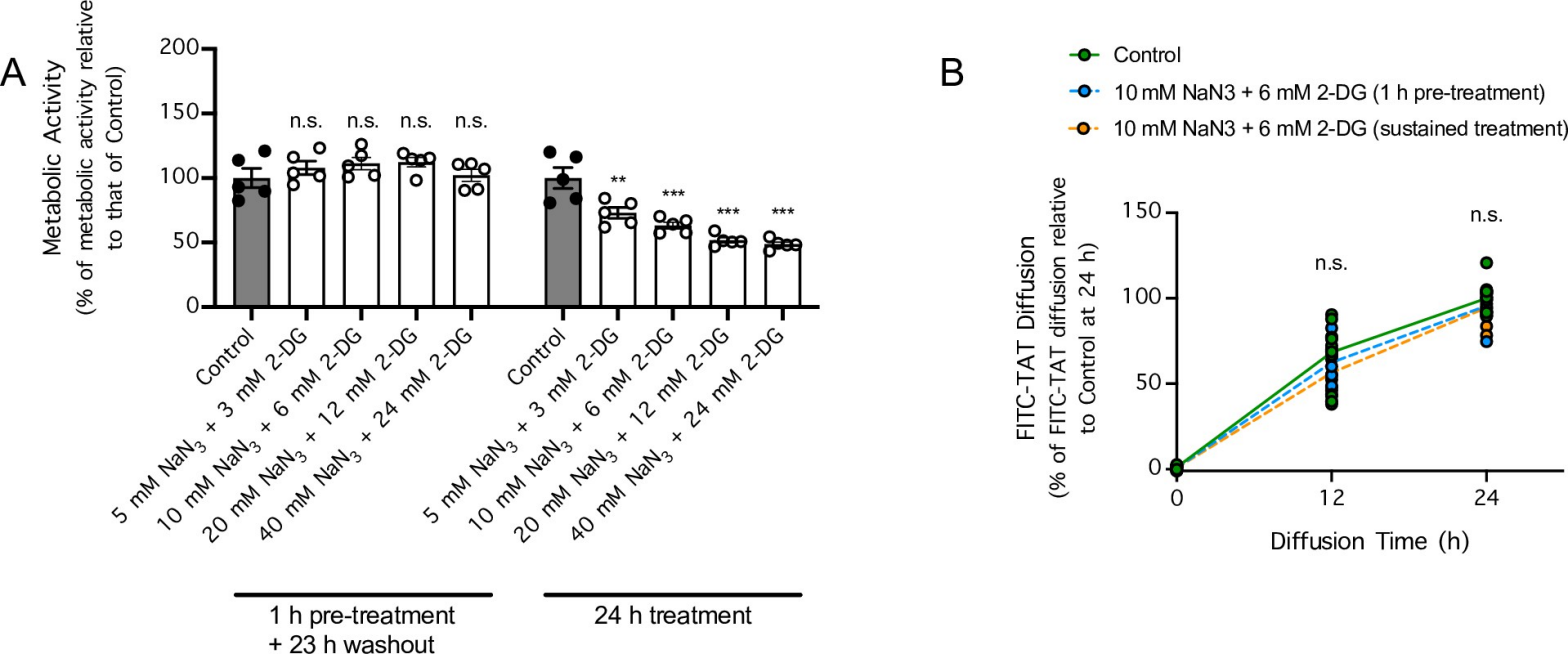
Cotreatment with sodium azide (NaN_3_) and 2-deoxy-D-glucose (2-DG) hinders cellular metabolic activity but not FITC-TAT diffusion across an endothelial monolayer. ***A***, Cellular metabolic activity levels in the bEnd.3 mouse brain endothelial cell line that were untreated (●) or cotreated with NaN_3_ (5–40 mM) and 2-DG (3–24 mM) for 1 h followed by a 23 h recovery period (○) or cotreated for 24 h without a recovery period (○). n = 5 cell cultures per group. ** *P* < 0.01, *** *P* < 0.001 for treated cultures compared to untreated cultures by 2-way ANOVA followed by Sidak’s multiple comparisons test. n.s. indicates no significant difference. ***B***, Diffusion of FITC-TAT (10 μM) across a monolayer of bEnd.3 cells that were untreated (●) or cotreated with NaN_3_ (10 mM) and 2-DG (6 mM) for 1 h followed by a 23 h recovery period (*gray circle*) or cotreated for 24 h without a recovery period (○), as determined 12 and 24 h post-diffusion onset via fluorescence spectrophotometry. n = 9 cell cultures per group. n.s. indicates no significant difference when the effect of treatments on FITC-TAT permeability was compared by 2-way repeated-measures ANOVA, matching [Tat-FITC] in the same culture medium over time.

Previous studies have shown that depletion of intracellular ATP level by co-treatment of cell cultures for 30–60 min with 10–15 mM NaN_3_, which inhibits oxidative phosphorylation, and 6–50 mM 2-DG, which inhibits glycolysis, can prevent cellular uptake of Tat-derived peptides and other related peptides [16, 18, 19]. Therefore, we next investigated whether a similar treatment regimen hinders the ability of FITC-TAT to cross a monolayer of brain endothelial cells. To identify a suitable treatment regimen that can persistently inhibit cellular metabolic activity for 24 h, we either pre-treated cultures of brain endothelial cells with 5–40 mM of NaN_3_ and 3–24 mM of 2-DG for 1 h, followed by a 23-h recovery period, or treated cultures for 24 h without a recovery period (Fig 2A). We found that pre-treating brain endothelial cells for 1 h with NaN_3_ and 2-DG failed to persistently decrease cellular metabolic activity following the 23-h washout period, whereas sustained treatment with NaN_3_ and 2-DG for 24 h decreased cellular metabolic activity in a concentration-dependent manner (Fig 2A). However, neither treatment regimens had an effect on the ability of FITC-TAT to cross the monolayer of brain endothelial cells (Fig 2B).

### Permeability of a monolayer of brain endothelial cell to Tat-derived peptide does not require receptor-mediated endocytosis

**Fig 3:**
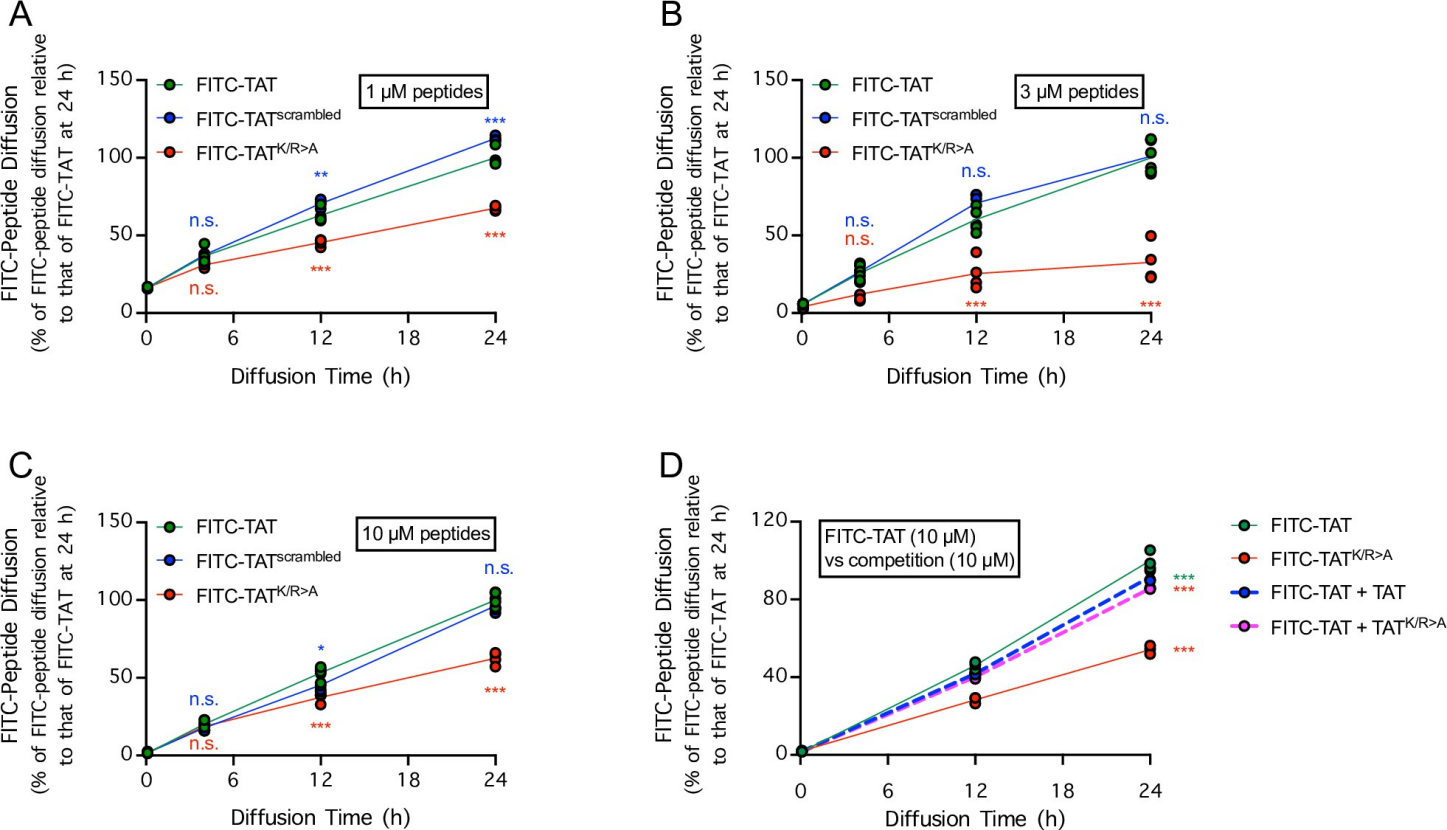
Diffusion of FITC-TAT across a monolayer of brain endothelial cells requires cationic amino acid residues but not competition for a receptor. ***A-C***, Diffusion of FITC-TAT (*green dots*), FITC-TAT^scrambled^ (*blue dots*), and FITC-TAT^K/R>A^ (*red dots*), after administration of 1 μM (***A***), 3 μM (***B***), or 10 μM (***C***) of the peptides, across a monolayer composed of the bEnd.3 mouse brain endothelial cell line was determined 4, 12, and 24 h post-diffusion onset via fluorescence spectrophotometry. n = 4 cell cultures per group. * *P* < 0.05, ** *P* < 0.01, *** *P* < 0.001, when FITC-TAT^scrambled^ or FITC-TAT^K/R>A^ was compared to FITC-TAT by 2-way repeated-measures ANOVA, matching [peptide] in the same culture medium over time followed by Sidak’s multiple comparisons test. n.s. indicates no significant difference. ***D***, Diffusion of FITC-TAT (10 μM) alone (*green dots*, *solid green line*), FITC-TAT^K/R>A^ (10 μM) alone (*red dots*, *solid red line*), or FITC-TAT (10 μM) coupled with either TAT (10 μM) (*green dots*, *dashed green line*) or TAT^K/R>A^ (10 μM) (*green dots*, *dashed red line*) across the bEnd.3 cell monolayer as determined 12 and 24 h post-diffusion onset via fluorescence spectrophotometry. n = 3 cell cultures per group. *** *P* < 0.001, compared to FITC-TAT alone by 2-way repeated-measures ANOVA, matching [peptide] in the same culture medium over time followed by Sidak’s multiple comparisons test.

Given the key role of cationic residues in cellular uptake of cell-permeable peptides [12–15], we next asked whether the ability of FITC-TAT to cross a monolayer of brain endothelial cells depend on its cationic residues or its specific amino acid sequence. In comparison to FITC-TAT, when FITC-TAT^scrambled^ (amino acid sequence: KRRRRYKRRQG, coupled with FITC attached to the peptide N-terminus), in which the amino acid sequence of TAT is scrambled, and FITC-TAT^K/R>A^ (amino acid sequence: YGAAAAAQAAA, coupled with FITC attached to the peptide N-terminus), in which lysine and arginine residues were replaced with alanine residues, were added to the upper chamber of Transwell inserts, FITC-TAT^scrambled^ but not TAT^K/R>A^ readily diffused across the monolayer of brain endothelial cells into the lower Transwell chamber (Fig 3A–3C). At 1 μM concentration, FITC-TAT^scrambled^ diffused across the monolayer at a marginally higher rate than FITC-TAT, whereas FITC-TAT^K/R>A^ diffused across the monolayer at a much lower rate than FITC-TAT (Fig 3A). At 3 μM concentration, FITC-TAT^scrambled^ diffused across the monolayer at a rate equal to that of FITC-TAT, whereas FITC-TAT^K/R>A^ diffused across the monolayer at a much lower rate than FITC-TAT (Fig 3B). At 10 μM concentration, FITC-TAT^scrambled^ diffuse across the monolayer at a marginally lower than FITC-TAT, whereas FITC-TAT^K/R>A^ diffused across the monolayer at a much lower rate than FITC-TAT (Fig 3C). These data suggest that the presence of arginine and lysine residues, but not the specific sequence by which the residues are ordered, dictated the ability of TAT-FITC to cross a monolayer of brain endothelial cells.

**Fig 4:**
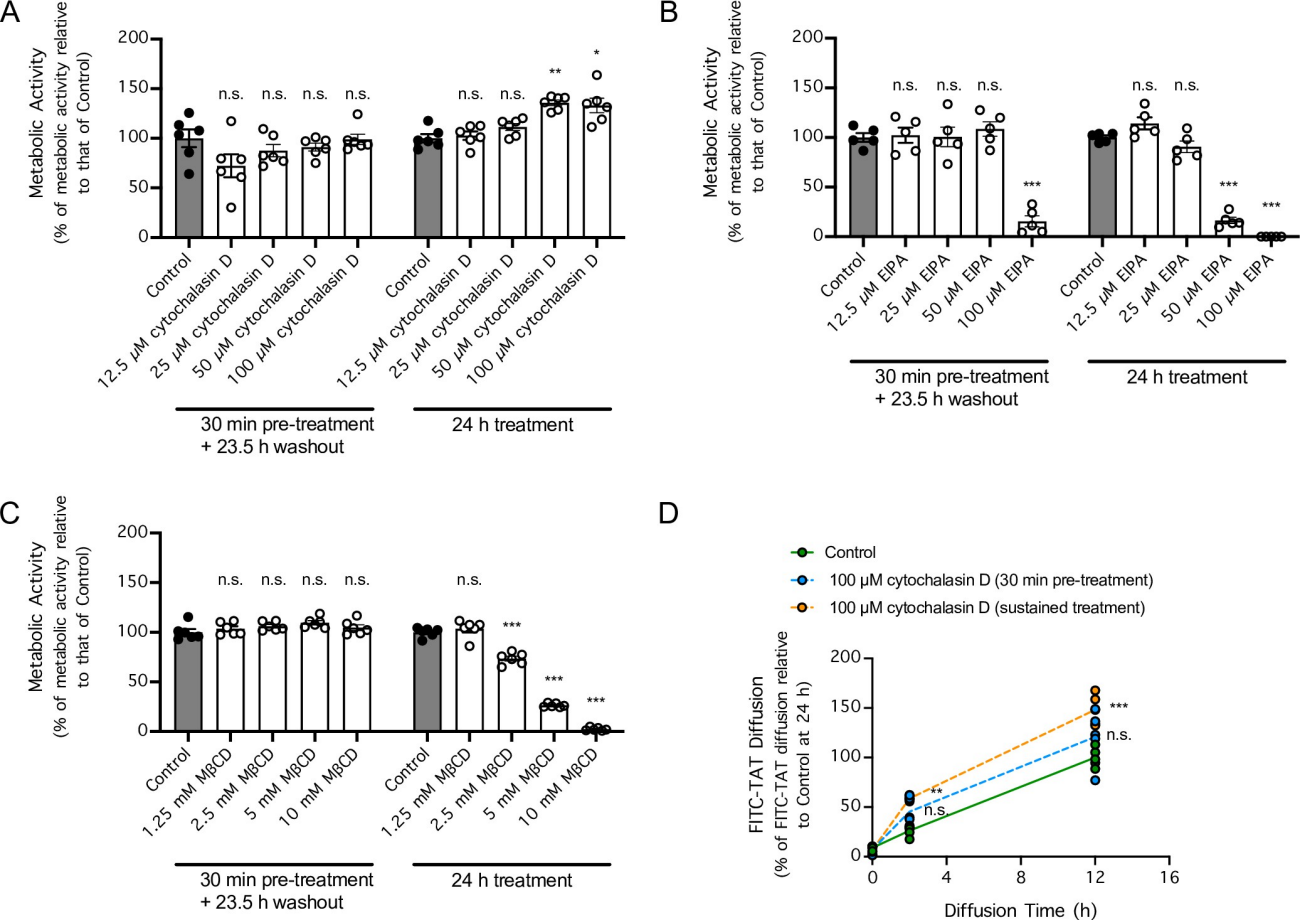
Effects of compounds that inhibit receptor-mediated endocytosis on cellular metabolic activity and diffusion of FITC-TAT across a monolayer of brain endothelial cells. ***A-C***, Cellular metabolic activity of the bEnd.3 mouse brain endothelial cells that were untreated (●) or treated (○) with cytochalasin D (***A***), 5-(N-Ethyl-N-isopropyl)-amiloride (EIPA) (***B***), or methyl-beta-cyclodextrin (MβCD) (***C***) for 30 min followed by a 23.5-h recovery period or treated for 24 h without a recovery period. n = 5 cell cultures per group. * *P* < 0.05, ** *P* < 0.01, *** *P* < 0.001 for treated cultures compared to untreated cultures by 2-way ANOVA followed by Sidak’s multiple comparisons test. n.s. indicates no significant difference. ***D***, Diffusion of FITC-TAT (10 μM) across a monolayer comprising bEnd.3 cells that were untreated (●) or treated with cytochalasin D (100 μM) for 30 min followed by a 23.5-h recovery period (*gray circle*) or treated for 24 h without a recovery period (○), as determined 2 and 12 h post-diffusion onset via fluorescence spectrophotometry. n = 5 cell cultures per group. *** *P* < 0.001 for the effect of treatments on FITC-TAT permeability compared by 2-way repeated-measures ANOVA, matching [Tat-FITC] in the same culture medium over time followed by Sidak’s multiple comparisons test. n.s. indicates no significant difference.

In light of the temperature-dependency and requirement for arginine and lysine residues of FITC-TAT, we next investigated whether FITC-TAT crossed the endothelial monolayer via receptor-dependent endocytosis. In contrast to the low permeability of the barrier to FITC-TAT^K/R>A^, the presence of non-FITC TAT or non-FITC TAT^K/R>A^ only marginally decreased the permeability of the monolayer barrier to FITC-TAT (Fig 3D). The lack of competition by non-FITC TAT suggests that the ability of FITC-TAT to cross the endothelial monolayer was not receptor-dependent. To further investigate whether receptor-mediated endocytosis is required for Tat-derived peptides to cross the endothelial monolayer, we asked whether inhibitors of endocytosis that had been previously shown to prevent cellular uptake of Tat-derived peptides [27] prevents FITC-TAT from crossing the endothelial monolayer. To ensure that any effect on the permeability to FITC-TAT was due to inhibition of endocytosis rather than potential secondary inhibitory effects on cellular metabolic activity, cultured brain endothelial cells were either pre-treated with cytochalasin D, EIPA, or MβCD for 30 min, followed by a 23.5 h recovery period, or were treated for 24 h without a recovery period (Fig 4A–4C). Interestingly, although pre-treatment of brain endothelial cells for 30 min with cytochalasin D, which inhibits endocytosis by disrupting actin filaments and has been shown to inhibit cellular uptake of Tat-derived peptides, had no effect on cellular metabolic activity following the 23-h washout period, sustained treatment with cytochalasin D for 24 h increased cellular metabolic activity in a concentration-dependent manner (Fig 4A). In contrast to the effect of cytochalasin D, treatment of brain endothelial cells with either EIPA or MβCD, both previously shown to inhibit cellular uptake of Tat-derived peptides, markedly decreased cellular metabolic activity (Fig 4B and 4C). Therefore, our data show that cytochalasin D was the most suitable inhibitor of endocytosis to use in our study because it did not cause a secondary effect of inhibiting cellular metabolic activities. Consistent with the increase in cellular metabolic activity induced by cytochalasin D, when brain endothelial cells were incubated with 100 μM cytochalasin D, FITC-TAT movement across the endothelial monolayer was increased rather than inhibited (Fig 4D). Altogether, these data demonstrated that receptor-mediated endocytosis was not required for FITC-TAT to cross the endothelial cell monolayer.

### The Tat-derived peptide disrupts the “in vitro BBB” formed by a monolayer of endothelial cells in a concentration-dependent manner

**Fig 5:**
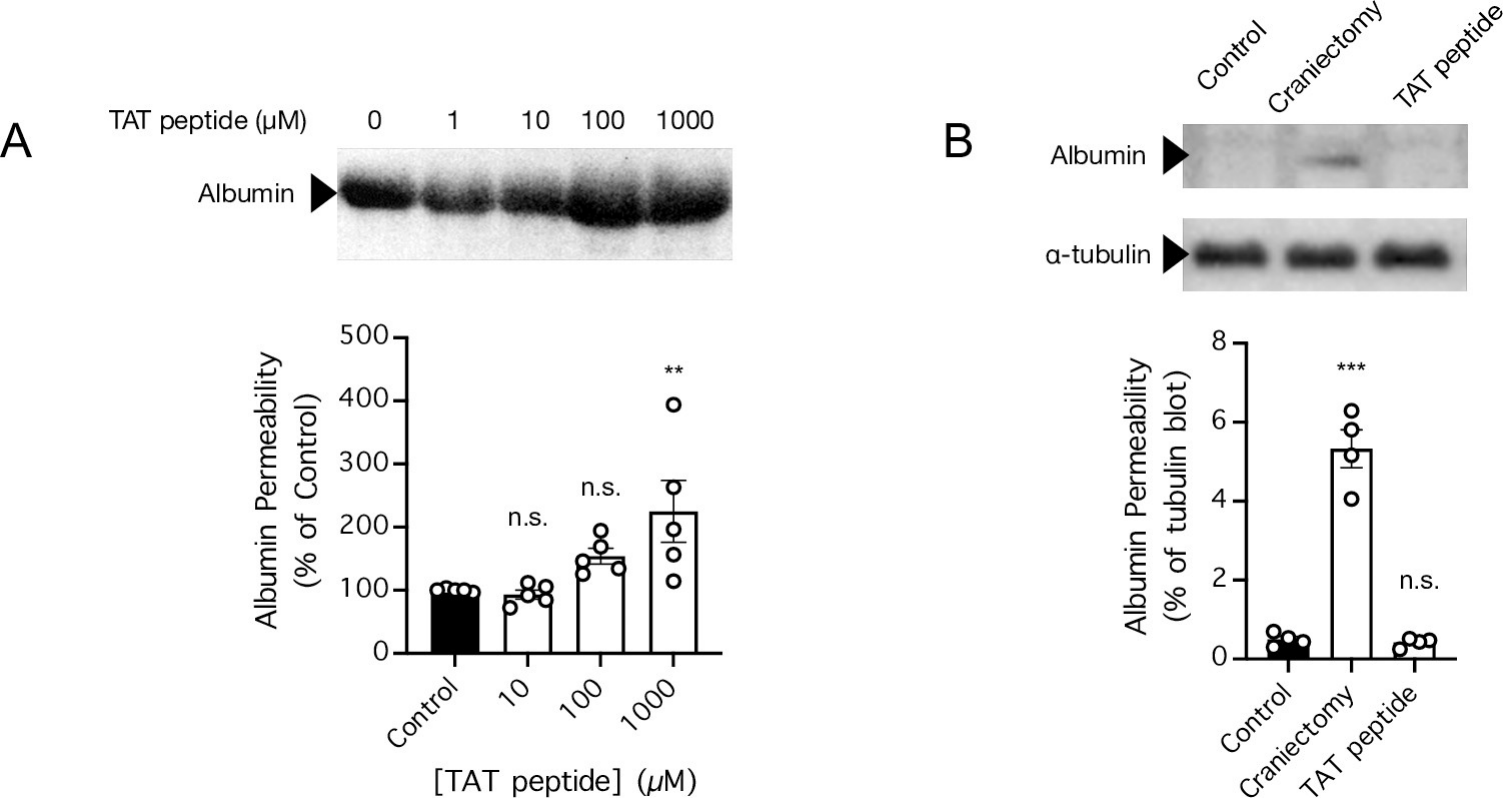
TAT administered at modest concentrations and doses exerted no effect on the permeability of a monolayer of brain endothelial cells *in vitro* and BBB permeability *in vivo*, respectively, but at a high concentration, TAT increased the permeability of the monolayer of brain endothelial cells *in vitro*. ***A*,** Diffusion of albumin across a monolayer of brain endothelial cells *in vitro* in the absence (control) or presence of TAT (10, 100, and 1000 μM) as determined by western blotting. n = 5 cell cultures per group. ** *P* < 0.01 for TAT-treated cultures compared to control by 1-way ANOVA followed by Sidak’s multiple comparisons test. n.s. indicates no significant difference. ***B*,** BBB permeability to albumin in control mice injected with vehicle (PBS, i.v. via tail vein) and mice subjected to craniectomy or injected with TAT (3 nmol/g, dissolved in PBS, i.v. via tail vein) was determined by western blotting. n = 4 mice per group. *** *P* < 0.001 for treated mice compared to control mice by 1-way ANOVA followed by Sidak’s multiple comparisons test. n.s. indicates no significant difference.

We next examined whether Tat-derived peptides crossed the monolayer of brain endothelial cells by disrupting the “*in vitro* BBB” prepared in this study. Interestingly, compared to the BBB permeability to other serum proteins and exogenous tracers, the permeability of BBB to albumin and albumin-binding tracers are the most reliable indicators for detecting a change in BBB permeability [56, 58]. Therefore, to examine whether TAT causes BBB disruption in our *in vitro* model, we measured the amount of albumin that crossed the monolayer of brain endothelial cells in the presence and/or absence of the TAT peptide (Fig 5A). Consistent with results reported in a previous study [54], we found that a 1000 μM concentration of the TAT peptide indeed significantly increased the amount of albumin that crossed the monolayer. However, at a 100 μM concentration, the effect of TAT on endothelial monolayer permeability to albumin was statistically nonsignificant, and at a 10 μM concentration, there was no noticeable difference between the control and TAT treatment groups on permeability to albumin (Fig 5A). Given that the 1000 μM concentration is unlikely to be found *in vivo* at treatment-level doses administered to animals or human subjects in previous animal studies and clinical trials, respectively, and that the more modest concentration of 10 μM [59] exerted no effect on the permeability of our “*in vitro* BBB”, our data suggest that BBB disruption was unlikely to be the mechanism by which Tat-derived peptides entered the brain in previous *in vivo* studies or in clinical trials [59–64]. Finally, to confirm the aforementioned conclusion, we examined whether the TAT peptide administered to mice at a commonly used dose (3 nmol/g) [59, 60, 65, 66] causes BBB disruption *in vivo* (Fig 5B). For the positive control, we subjected a group of mice to craniectomy, which has recently been shown to reliably increase BBB permeability [56]. Consistent with the conclusion based on our *in vitro* data, the administration of the TAT peptide (3 nmol/g, injected intravenously via tail vein) failed to increase BBB permeability to albumin in mice *in vivo* (Fig 5B); in contrast, craniectomy greatly increased BBB permeability to albumin (Fig 5B). Our *in vitro* and *in vivo* data together showed that the TAT peptide, at a dose commonly used *in vivo*, did not disrupt the BBB.

## Discussion

Biologics, including therapeutic peptides and proteins, have been at the forefront of pharmaceutical development in recent years [67]. However, there are several disadvantages to the use of biologics in comparison to the use of conventional small-molecule drugs. For example, biologics are typically not orally available due to their possible degradation by the gastric environment. Also, biologics often need to be kept in specially designated formulations that could cause pain at the site of injection [67, 68]. Another major obstacle associated with the pharmaceutical development of biologics is the low permeability of biological barriers, including cellular plasma membranes and vascular barriers such as the BBB, to these therapeutic agents. Therefore, to enable noninvasive treatment of brain diseases, the recent decades have seen growing development of TAT-linked biologics, especially TAT-linked peptides, that are used to treat brain diseases in preclinical animal models and in clinical trials [64, 69, 70].

To improve the potency of TAT-linked peptides for treating brain diseases, it is crucial to better understand the mechanisms by which these peptides cross the BBB. In this study, the ability of FITC-TAT to cross a monolayer of brain endothelial cells, an *in vitro* model of the BBB [55], was substantially decreased when the temperature was lowered from 37°C to 4°C. This finding suggests that BBB permeability to FITC-TAT is temperature-dependent. This temperature dependency aligns with the general notion that pathological changes to BBB permeability in model animals are temperature-dependent [71]. Notably, in early studies, TAT and other related peptides were mistakenly believed to cross cell membrane in a temperature- and cellular metabolic activity-independent manner [11, 13–15]. However, these early findings were caused by experimental artifacts related to cell fixation and flow cytometry analysis [16, 17]. It is now widely accepted that TAT-linked peptides, at least when administered at commonly used doses and concentrations, enter cells in a temperature-dependent manner and that cellular uptake of these peptides can be inhibited by the depletion of cellular metabolic activity via NaN_3_ treatment [16, 18–21]. In comparison, although reducing the temperature decreased the cellular metabolic activity of brain endothelial cells and inhibited the ability of FITC-TAT to cross the monolayer of brain endothelial cells in this study, decreasing the cellular metabolic activity *per se* with NaN_3_, without decreasing the temperature, failed to inhibit the ability of FITC-TAT to cross the monolayer of brain endothelial cells. Therefore, these findings altogether suggest that both cellular uptake of Tat-linked peptides and BBB permeability to FITC-TAT are temperature-dependent, but only the former can be inhibited by depleting cellular metabolic activity via NaN_3_ treatment.

Previous studies have suggested that cellular uptake of TAT-linked peptides depends on the cationic residues in the peptides, and consistent with this supposition, the amount of polyarginine oligopeptides taken up into cells was similar to or even greater than that of TAT-linked peptides [12–15]. In this study, the amount of FITC-TAT that crossed the monolayer of brain endothelial cells was substantially decreased when the lysine and arginine residues were replaced with alanine residues. Notably, the substitution of amino acid residues can interfere with the peptide structure of TAT, thereby preventing the peptide from binding to its biological target. To test whether this was the case, we also compared the endothelial permeability of FITC-TAT to the permeability of a FITC-TAT^scrambled^ peptide. In this experiment, we found that the endothelial cell monolayer permeability to FITC-TAT^scrambled^ was no different from the permeability to the native FITC-TAT. Taken together, these results suggest that, similar to the mechanism underlying its cellular uptake, the BBB permeability to TAT depends on the peptide cationic domain, rather than the specific sequence in which the residues are ordered.

Multiple studies have reported that cellular uptake of TAT involves receptor-mediated endocytosis as well as other forms of endocytosis [19–21, 23, 25–34]; nevertheless, until the present study, there has been little evidence to show whether BBB permeability to TAT is also receptor-dependent. First, we found in this study that the ability of FITC-TAT to cross the monolayer of brain endothelial cells was only marginally inhibited in the presence of equimolar concentration of a non-FITC TAT peptide, suggesting a lack of competition for receptor-binding. Furthermore, the same marginal inhibition was also observed with equimolar concentration of a non-FITC TAT^K/R>A^; therefore, the marginal inhibitory effect of non-FITC TAT was not due to competition between FITC-TAT and non-FITC TAT peptides for receptor-binding. Second, we report in this study that cytochalasin D, which has been previously shown to inhibit the cellular uptake of TAT by inhibiting receptor-mediated endocytosis, failed to inhibit the ability of FITC-TAT to cross the monolayer of brain endothelial cells. Taken together, our data suggest that, in contrast to the ability of cells to take up TAT and related peptides, BBB permeability to TAT is not receptor-dependent.

Given that the full-length Tat protein can disrupt the BBB by increasing vascular endothelial permeability [41, 42], inducing endothelial cytotoxicity [43–47], and by modulating the expression and cellular localization of tight junction proteins needed to maintain adhesion of the brain endothelial cells to each other [48–52], at least two studies have explored the possibility that BBB permeability to TAT is achieved via induction of BBB disruption [53, 54]. However, their results were contradictory, with one study showing that TAT increased brain endothelial cell monolayer permeability [54], and the other study showing that TAT did not increase brain endothelial cell monolayer permeability [53]. In this study, we reconciled and reproduced the results of both studies to show that a modest concentration (10 μM) of TAT exerted no effect on the permeability of a monolayer of endothelial cells to albumin, but a very high concentration (1000 μM) of TAT substantially increased the endothelial permeability to albumin. Notably, our result is in line with recent experiments showing that, at a higher concentration and under certain experimental conditions, cellular uptake of TAT can be achieved via transient focal deformation of certain regions in the cell membrane [24, 29, 32, 35–40]. Given that the very high concentration (1000 μM) required for disrupting permeability across a monolayer of brain endothelial cells is unlikely to be achieved *in vivo* when administered at doses used in previous preclinical animal studies and clinical trials [59–64], we reasoned that at commonly used doses, TAT cannot cause BBB disruption *in vivo*. To test our hypothesis, we subjected mice to either craniectomy, a method proven to disrupt the BBB [56], or injection of TAT at a the commonly used dose of 3 nmol/g [59, 60, 65, 66]. Indeed, we found that craniectomy reliably increased BBB permeability to serum albumin in mice, whereas TAT at the commonly used dose had no effect on BBB permeability. Therefore, although at a very high concentration, TAT can disrupt a monolayer of brain endothelial cells *in vitro*, the mechanism underlying this disruption is probably not the same mechanism by which TAT-linked peptides have been delivered to the brain in preclinical studies *in vivo* and in clinical trials.

In conclusion, our data demonstrated that the mechanism by which modest concentrations of TAT cross a monolayer of brain endothelial cells differs from the mechanisms by which TAT enters into cells. Although both mechanisms are temperature-dependent, in contrast to the cellular uptake of TAT, TAT movement across a monolayer of brain endothelial cells cannot be inhibited by energy depletion induced by NaN_3_ treatment and does not involve receptor-mediated endocytosis. Interestingly, at a very high concentration, TAT disrupted the permeability of the brain endothelial cell monolayer, similar to its ability to enter cells by causing focal deformation of the plasma membrane at higher concentrations. Future studies should focus on how TAT at modest concentrations that are typically met in preclinical *in vivo* animal studies and clinical trials can cross the BBB.

## Supporting information

S1 Data (XLSX)

S1 Raw images (PDF)
