# Supplementary figures and images for: TAT peptide at treatment-level concentrations crossed brain endothelial cell monolayer independent of receptor-mediated endocytosis or peptide-inflicted barrier disruption
Source: PLoS One. 2023 Oct 11;18(10):e0292681. doi: 10.1371/journal.pone.0292681 (PMC10566733; doi:10.1371/journal.pone.0292681)

10 ul/well

Time (hr)

24

TAT ( $\mu$ M)

0

10

100

1000

Blot: albumin

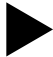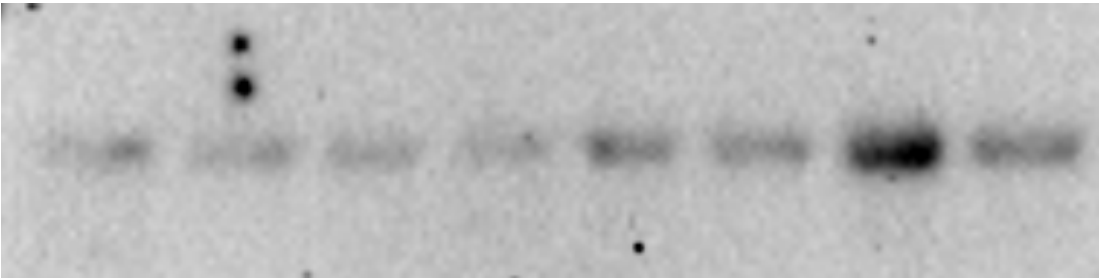

5 ul/well

TAT ( $\mu$ M)

0

10

100

1000

Blot: albumin

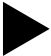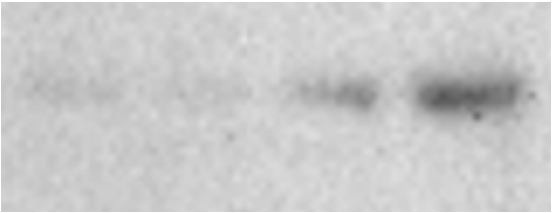

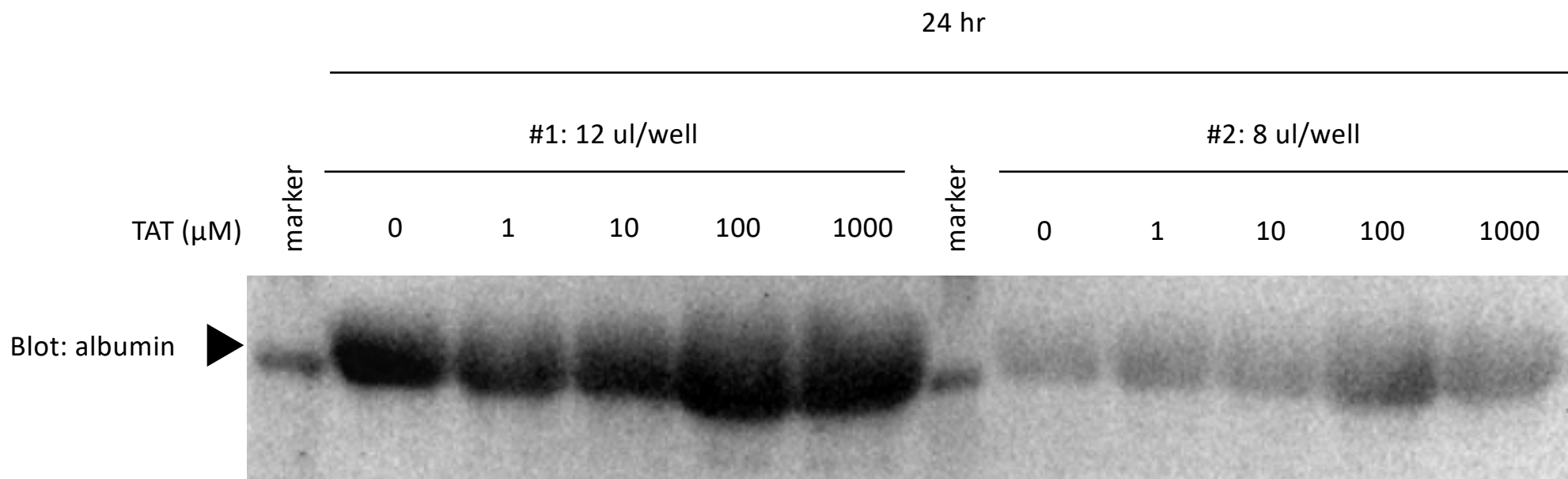

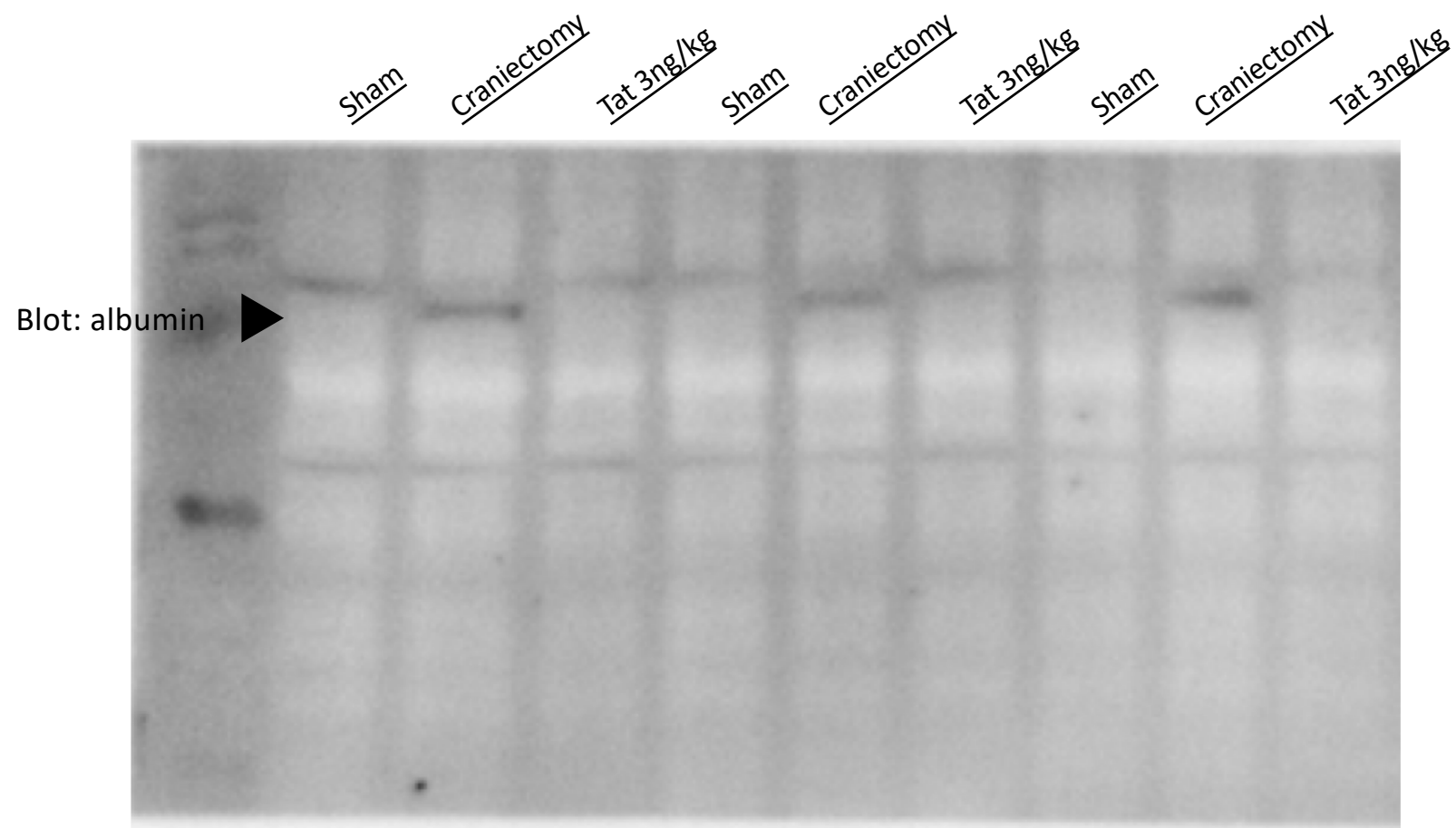

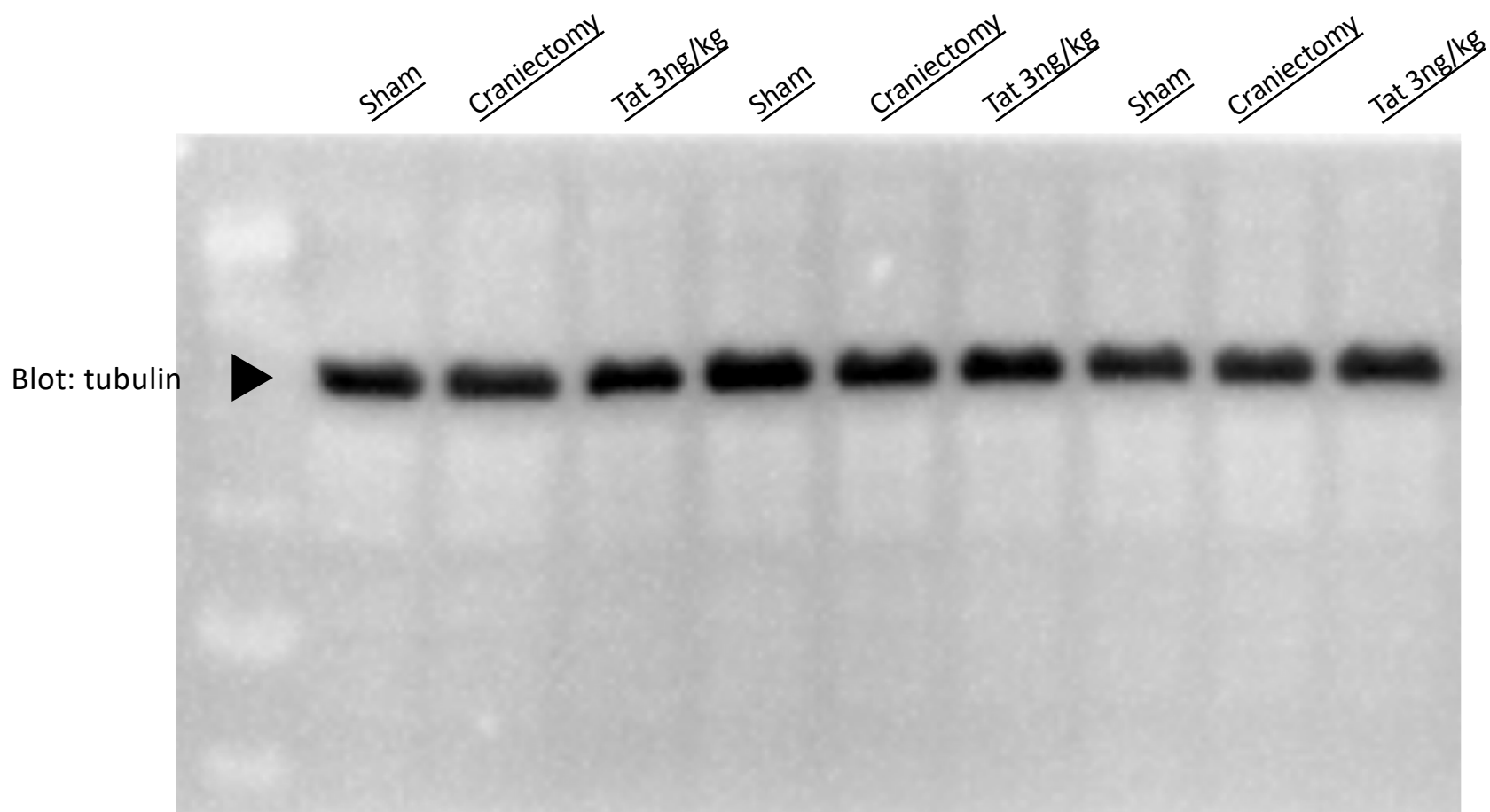

Supplement: S1 Raw images — (PDF) [file pone.0292681.s002.pdf]
